# How Large, Decayed, and Moist Must Rotting Logs Be to Act as Thermally Buffered Microhabitats in Temperate Eastern United States Forests?

**DOI:** 10.1002/ece3.73591

**Published:** 2026-05-06

**Authors:** Ryan T. Phillips, Ryan C. Garrick

**Affiliations:** ^1^ Department of Biology University of Mississippi Oxford Mississippi USA

**Keywords:** coarse woody debris, deadwood, forest management, microclimate, saproxylic, southern Appalachian Mountains

## Abstract

Deadwood is an essential habitat for forest biodiversity, yet coarse woody debris (CWD) is declining owing to silvicultural practices that impede recruitment (e.g., clearcutting, short logging rotations), and/or forest sanitization that prevents retention (e.g., removal of “waste wood”). Furthermore, connections among studies examining the ecological roles of CWD as potential refugia for saproxylic organisms susceptible to heat and desiccation, or cold, have been hampered by inconsistent thresholds used to define “large‐diameter” logs. Here we measured temperature inside and outside rotting logs over four seasons in temperate montane Southern Appalachian forests, and assessed relationships between several metrics of thermal buffering with log diameter, decay stage, and moisture content. Our analyses showed that the microclimates of larger diameter logs exhibit significantly decreased temperature ranges in spring and fall, and increased minimum temperatures in winter and on extreme cold days. Qualitatively, logs with diameters of at least 25–30 cm provided modest to considerable thermal buffering (depending on the metric), and this may represent a useful threshold for targeted enhancement of CWD habitats. However, there was no detectable reduction of maximum temperatures in summer or on extreme warm days, suggesting that, in natural temperate mixed pine‐oak forests in the eastern United States, a key benefit that saproxylic organisms gain from living in rotting logs may be avoidance of cold stress. Neither decay stage nor moisture content were significant predictors of thermal buffering in any season. Ultimately, larger diameter logs provide important microhabitats, and management strategies that prioritize their creation and retention should have far‐reaching benefits for biodiversity in forests.

## Graphical abstract

This study addresses the ecological role of coarse woody debris (CWD) as a thermally buffered microhabitat for saproxylic organisms in temperate eastern US forests. We measured internal and external log temperatures across four seasons in Southern Appalachian forests to test how log diameter, decay stage, and moisture influence thermal buffering. Our results show that larger logs (≥ 25–30 cm) substantially reduce temperature variability in spring and fall and raise minimum temperatures in winter, but do not reduce summer maxima.

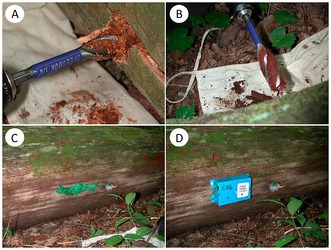

## 1 Introduction

Deadwood is an important carbon pool in forests, storing 8% of global forest carbon, and it is considered an essential microhabitat for 25% of forest biodiversity (Pan et al. 2011; Stokland et al. 2012; Martin et al. 2021). Saproxylic organisms, which rely on deadwood to complete their life cycle (Speight 1989), are a phylogenetically and functionally diverse group that includes detritovores, fungivores, omnivores, and carnivores, yet they are an overlooked component of forest biodiversity (Siitonen 2001; Grove 2002a). Deadwood‐dependent organisms are key ecosystem service providers that contribute to macro‐ and micronutrient cycling by mechanically fragmenting and enzymatically digesting cellulose and hemicellulose in wood, returning the carbon, nitrogen, and other nutrients stored in logs to an accessible state (Ausmus 1977; Ulyshen and Wagner 2013; Ulyshen 2016). Through chewing and burrowing, wood‐feeding invertebrates create galleries within logs that serve as conduits for microbial colonization, which results in further increases in surface area of internal cavities (Maser et al. 1988; Van Lear 1993). In turn, this increased structural complexity makes rotting logs suitable for taxa that are facultatively associated with this microhabitat (Harmon et al. 1986; Graf et al. 2022).

As a group, saproxylic organisms are threatened by human activities that reduce the spatial and temporal continuity of coarse woody debris (CWD; Schiegg 2000; Seibold et al. 2015). This includes land clearing for agriculture, silvicultural practices such as clear‐cutting, firewood collection, and other forest sanitization practices that treat deadwood as waste wood (Grove 2002a). For example, managed forests in Fennoscandia have been estimated to contain at least 90% less CWD than their unmanaged, old growth counterparts (Siitonen 2001), a pattern broadly consistent with studies from other temperate regions showing reduced deadwood availability under intensive management (Harmon et al. 1986). This extensive volume reduction is accompanied by an overall reduction in the mean diameter of remaining CWD. In the case of clearcutting, this reduction in the mean diameter occurs because logging rotations are relatively short, such that regrowth trees do not reach large diameters before being harvested again (Van Lear 1993).

Forest management practices that explicitly consider the needs of saproxylic species are well‐established in Europe, and often emphasize the importance of large‐diameter CWD, which includes logs on the forest floor, as well as standing dead trees (i.e., snags). For example, Key and Ball (1993) questioned the value of retaining small‐sized deadwood, and instead advocated for maintaining as much large deadwood as possible. Likewise, in a United Kingdom Forestry Commission report, Humphrey et al. (2002) stated that large‐diameter deadwood is most valuable. This sentiment is not without merit, as research has shown that large‐diameter logs support a greater richness and abundance of saproxylic assemblages than small diameter logs (Grove 2002a). Furthermore, large logs may be essential habitat for certain at‐risk and high conservation priority taxa. For example, a study in Sweden found that four times more IUCN Red‐Listed saproxylic invertebrate species occur in large diameter logs than in smaller logs (Jonsell et al. 1998). However, the notion of what constitutes a “large‐diameter log” or “CWD” varies considerably. For example, the United States Forest Service definition of CWD specifies that the diameter (at the point of intersection of a line transect) must be > 7.6 cm (USFS 2018), yet ≥ 15.0 cm has been adopted by the United Kingdom Forestry Commission (Hodge and Peterken 1998), and ≥ 20.0 cm has occasionally also been used (Humphrey et al. 2002). This inconsistency highlights an important knowledge gap: how large rotting logs must be to provide thermally buffered microhabitats, a key component of habitat suitability for many forest floor and saproxylic organisms, as microclimatic stability strongly influences the distribution and persistence of temperature‐ and moisture‐sensitive taxa (e.g., Scheffers et al. 2014).

Although thermal tolerance data for saproxylic invertebrates are limited, the available studies suggest that Critical Thermal maxima (CTmax) are typically quite high (40°C–49°C; Barnes et al. 2022; Lawhorn and Yanoviak 2022), well above temperatures in forest floor environments. Conversely, lower thermal limits and sublethal performance constraints occur within the range of naturally experienced conditions. For example, Cox et al. (2020) reported a Critical Thermal minimum of ~4°C and sprint speed performance optima near 34°C for a wood‐dwelling centipede from mixed pine‐oak forests in the southeastern United States, indicating potential sensitivity to cold conditions. These findings suggest that microclimatic buffering—particularly of low temperatures and rapid fluctuations—may be more relevant than avoidance of extreme heat, although direct links between microclimate and organismal performance remain poorly resolved. That said, research on soft‐ and hard‐bodied saproxylic invertebrates from temperate sclerophyll forests in southeastern Australia has shown that other ecophysiological traits such as rates of water loss and respiration increase between 7% and 13% per 1°C increase in temperature (Schmuki et al. 2007; Woodman et al. 2007). As such, sublethal warm temperatures likely impose considerable costs upon organismal performance and/or expenditures of energy.

Rotting logs are known to be thermally buffered from ambient temperatures, and Kluber et al. (2009) suggested that log diameter is a potential determinant of overall buffering capacity. However, since diameter has previously been treated as a categorical variable with a somewhat arbitrary threshold between “small” versus “large” logs, it can be difficult to connect inferences across different studies. Saproxylic organisms are often sensitive to high temperatures and prone to desiccation, and thus rely on the stable microclimates that rotting logs provide (Garrick et al. 2007; Schmuki et al. 2007; Woodman et al. 2007; Weldon et al. 2013). While some studies have suggested that logs at advanced stages of decay are more thermally buffered (e.g., Barnes et al. 2022; Lawhorn and Yanoviak 2022), in the absence of replication, it is unclear how robust these conclusions are. Beyond log size and decay stage, other factors may also be important. For example, the interior environment of rotting logs is usually quite moist (Romo et al. 2019), and this can not only affect thermal properties, but also offer protection from desiccation—particularly for soft‐bodied saproxylic invertebrates (Sunnucks et al. 2006; Garrick et al. 2008). From a conservation perspective, it is important to understand what underlies the variation in thermal buffering within and among rotting logs, especially since log attributes can vary owing to different forest management histories. Specifically, we need to know how decayed and how moist rotting logs need to be to act as thermally buffered microhabitats for the biota that depend on deadwood.

Here we focus on temperate forests in the Southern Appalachian Mountains, in the southeastern United States. This region is a globally recognized center of endemism (e.g., salamanders; Rissler and Smith 2010), and harbors many as‐yet undescribed forest floor and deadwood‐associated invertebrate species (e.g., Carlton and Bayless 2007; Marek 2010; Garrick et al. 2018; Hennen et al. 2022). This is cause for concern given the region's history of intensive timber extraction. Indeed, nearly all forests have been harvested at least once since the mid‐1800s, and today, National Parks (i.e., areas that offer maximum protection of biodiversity) account for < 3% of the region, whereas > 80% is privately owned, and intensive timber extraction is on‐going (SAMAB 1996). Thus, there is an urgent need for actionable data‐driven conservation recommendations, and ideally, they should be applicable to both public and private lands (Grove 2002a). This can be challenging, given the contrasting management objectives, forest age structure, and deadwood inputs in minimally managed public lands versus silvicultural plantations. However, privately owned forest lands used for hunting or other recreational activities, including areas under conservation easement, represent an often underappreciated opportunity for conservation of deadwood.

Here we aimed to identify general patterns in thermal dynamics of CWD and provide a foundation for future hypothesis‐driven studies. Furthermore, by characterizing when and how the microclimatic environment within logs may become stressful, outcomes of this work will be directly useful for guiding the design of future functional ecology experiments, where an understanding of “natural” thermal regimes is essential. To address knowledge gaps about how large, decayed, and moist rotting logs must be to act as thermally buffered microhabitats for saproxylic fauna in temperate mixed pine‐oak forests, we measured the diameter, decay stage, and gravimetric moisture content of logs from publicly owned lands in the Southern Appalachian Mountains. The focal study regions are managed for multiple uses including conservation, recreation, and in some locations, timber production, and are generally natural, uneven‐aged regrowth forest. We then tested the ability of these variables to predict three ecologically relevant components of thermal buffering of internal deadwood microhabitats, relative to the external environment: (1) reduction of average daily maximum temperatures in summer, (2) increase of average daily minimum temperatures in winter, and (3) decrease in the range of temperatures experienced in spring or fall. Findings of this study are discussed in the context of forest management practices that advance the conservation of saproxylic organisms in the Southern Appalachian Mountains and elsewhere, including suggestions for surveying, retaining, and augmenting CWD habitats.

## 2 Materials and Methods

### 2.1 Study Area

**FIGURE 1:**
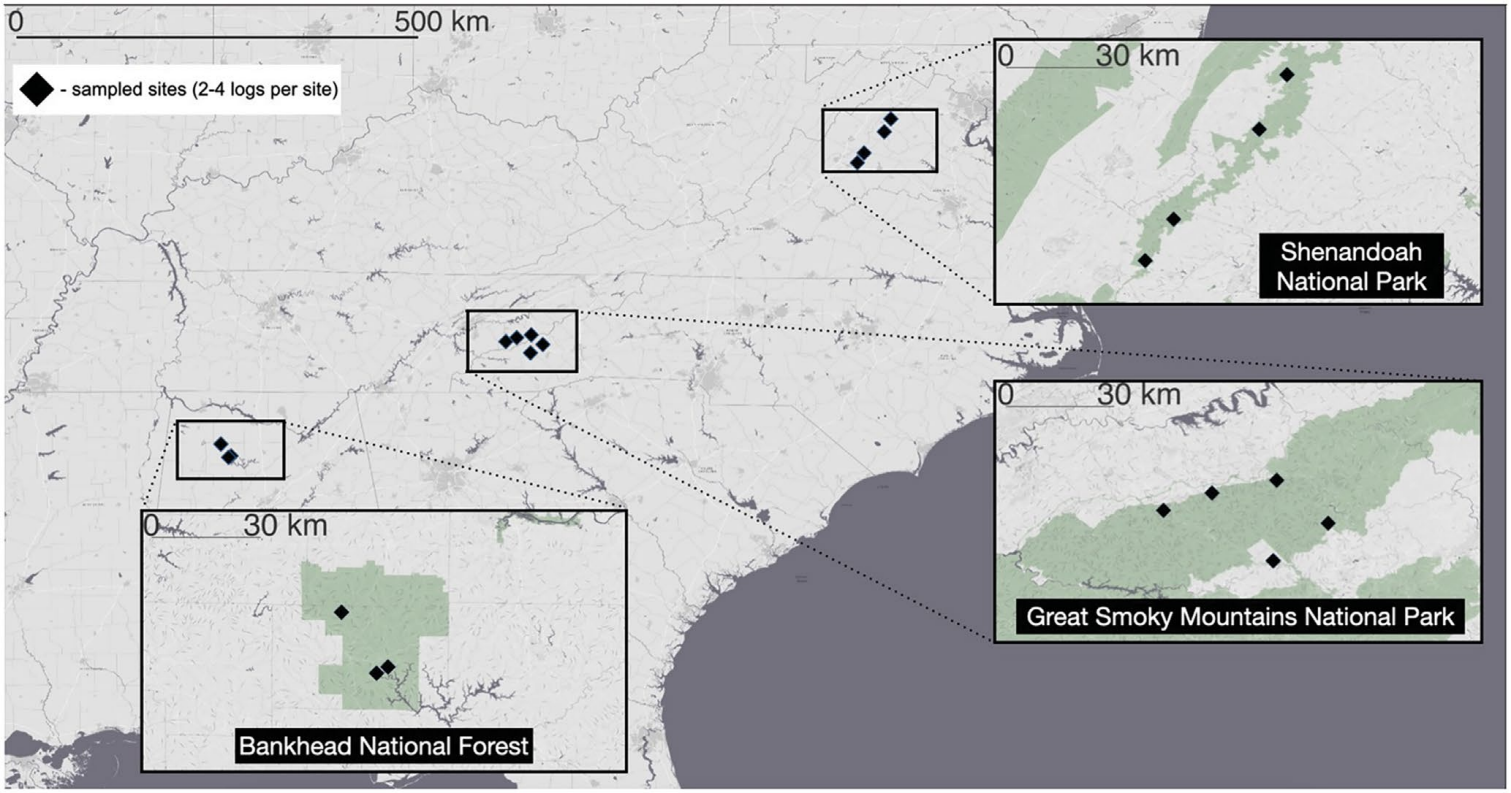
Map showing distribution of three forest regions in the Southern Appalachian Mountains (boxes), and local sites within them (i.e., 150 m × 150 m areas that contained multiple focal logs; black diamonds). Insets: Higher spatial resolution within each forest region, and shading distinguishes National Forest or National Park (green) from nonpublic lands.

Fieldwork was conducted under permits from USDA Forest Service and US National Park Service (GRSM‐2015‐SCI‐2242 and SHEN‐2015‐SCI‐0020). Between July 2015 and June 2017, we gathered size, decay stage, moisture content, and temperature data from rotting logs in three forest regions across the Southern Appalachian Mountains: Bankhead National Forest, Alabama; Great Smoky Mountains National Park, Tennessee; and Shenandoah National Park, Virginia (Figure 1). Within each region, focal logs were distributed across three to five local sites (each defined as a 150 m × 150 m area containing multiple focal logs).

Bankhead National Forest is a mixed pine–oak system composed of bottomland hardwood, forested wetlands, and upland pine (shortleaf and loblolly), interspersed with agricultural fields and pastures on interdigitated private lands. It is relatively low‐lying (~200–300 m) with warm summers and high precipitation, with mean monthly temperatures ranging from ~5°C–10°C in winter to ~25°C–27°C in summer (USFS 2004; PRISM Climate Group 2026). The forest is managed by the U.S. Department of Agriculture Forest Service for multiple uses, including wildlife conservation, recreation, and timber extraction, and includes silvicultural plantations as well as protected wilderness areas with limited old‐growth forest (Burgess and Garrick 2020). Much of the region was heavily logged and degraded in the early 1900s and has since been reforested and actively managed (USFS 2004). The Great Smoky Mountains and Shenandoah National Parks are both temperate hardwood forest systems managed by the U.S. Department of the Interior National Park Service for conservation and recreation, with no active timber harvest. These two regions experienced extensive logging in the early 1900s, but since park establishment in the mid‐1930s, they have been protected landscapes (USGS 2026a, 2026b). These two national parks are characterized by humid climates, with temperatures ranging from below freezing at higher elevations in winter to ~20°C–27°C in summer, depending on elevation (PRISM Climate Group 2026), strong elevational gradients, and structurally complex forests that include eastern hemlock in mesic habitats (USGS 2026a, 2026b). However, they differ in several notable ways. Great Smoky Mountains National Park supports a broader range of forest types, including cove hardwood, northern hardwood, oak–pine, and high‐elevation spruce–fir forests, and spans a much wider elevational gradient (~250–2025 m), resulting in pronounced climatic variation and very high precipitation (GSMIT 2017). In contrast, Shenandoah National Park is dominated by oak–hickory forests with mixed mesophytic hardwoods in sheltered sites and pine on drier ridges, spans a more moderate elevational range (~180–1235 m), and exhibits less climatic variability (NPS 2007). Additionally, while Great Smoky Mountains National Park contains extensive mature forest and some of the most substantial tracts of old‐growth forest in the eastern United States, Shenandoah National Park forests are largely second‐growth, recovering from historical logging, agriculture, and settlement, with comparatively limited old‐growth remnants (USGS 2026a, 2026b).

### 2.2 Temperature Data

**FIGURE 2:**
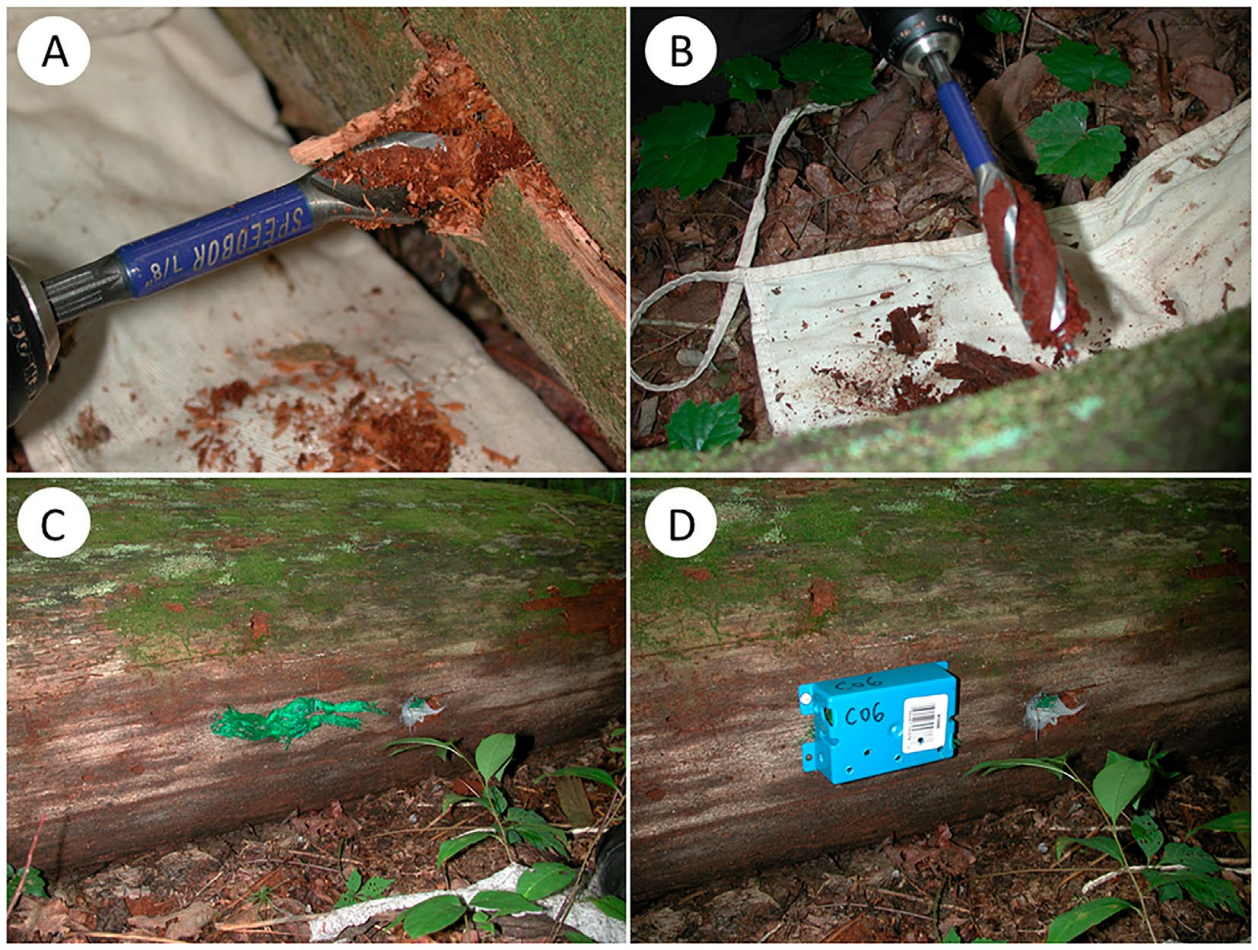
Exemplar images illustrating the field procedure used to deploy Thermochron iButtons inside and outside of focal rotting logs. (A) drilling a 10 cm deep hole into the side of a log; (B) collecting rotting woody debris for subsequent repacking of the drilled hole; (C) positions of external (left) and internal (right) iButtons; and (D) placement of plastic cover to protect external iButtons from direct sunlight and animal interference.

Temperature data were collected using Thermochron iButtons (model # DS1921G, temperature range −40°C to 85°C) with date and time synchronized, and mission set to record at 4‐h intervals. Note that iButton accuracy (±0.5°C) reflects maximum single‐measurement error, but because buffering metrics were derived from repeated paired measurements over time (see below), random error is expected to average out and not affect internal–external contrasts. To reduce the likelihood of device failure under field conditions, all iButtons were waterproofed by applying a thin line of clear superglue where the two sections of metal casing meet (i.e., around the perimeter only). Roznik and Alford (2012) reported a minimal influence on temperature readings when the same model of iButton was fully coated with a clear plastic dip, and so our pretreatment is unlikely to have led to systematic bias. For each focal rotting log, internal temperature data were obtained by drilling a 10 cm deep hole into the side, parallel to the ground, at the midpoint of the structurally contiguous section of the chosen log (i.e., between its two distinct ends/severe breaks) using a cordless drill (Irwin woodboring tri‐flute drill bit, 22.2 mm diameter, model # 3041005). Hole depth was standardized at 10 cm for all logs to ensure comparability among samples and to target interior wood. Even in the smallest log (15 cm diameter, see Results), this depth still captured inner wood well removed from the outer surface. To facilitate later retrieval, iButtons were placed in a thin flexible nylon netting sleeve (bar mesh size 8.5 mm, stretch mesh size 13.5 mm) prior to insertion into the drilled hole. The hole was repacked with the original rotting woody debris, then a cork was hammered into the opening until flush with the outer surface, and silicone was used to seal the cork/hole junction. For the same rotting logs that internal temperature data were obtained, external data were collected by attaching iButtons (in the same nylon netting sleeve as above) to the outside of the log. These external iButtons were laterally offset from the cork/hole by ~20 cm, and they were shielded from direct solar radiation and animal interference using ventilated covers designed to allow airflow and minimize heat buildup, thereby approximating shaded ambient conditions. The covers were constructed from plastic (Carlon PVC shallow flanged electrical switch box, model B108R‐UPC), each with 14 large (5 mm diameter) holes drilled in all surfaces except for the upper surface (Figure 2). Nonetheless, we acknowledge the potential for some residual warming. All internal and external iButtons were deployed as redundant pairs in case of device failure or malfunction.

### 2.3 Rotting Log Size, Decay Class, and Moisture Content

Log diameter, decay stage, and moisture content were measured as continuous (or ordinal) predictors in an observational framework, reflecting naturally occurring variation rather than experimentally manipulated factors. Following temperature data collection, a 0.915 m (i.e., 3 ft) long section of each focal log that contained the iButtons was removed using a chainsaw. The circumference (C) at mid‐point was determined using a tape measure and then converted to diameter (d) by dividing by 3.14, assuming a circle. Following Barclay et al.'s (2000) classification scheme, log decay class (on a scale of 1–5, with 5 representing the most advanced decay) was estimated by examining the cross‐section profile of each end of the removed section, as well as other general characteristics of the log in situ (e.g., how intact the exterior remained). The gravimetric method was used to quantify moisture content of each focal rotting log. Briefly, a 50 mL centrifuge tube was filled with decomposing wood sampled from a location immediately surrounding internal iButtons, capped, and then sealed with parafilm to prevent evaporative water loss during transportation. Subsequently, wet weight (WW) was measured (in grams to two decimal places) using a digital scale. The wood sample was then placed in a lab drying oven at 70°C for 72 h, and reweighed to obtain dry weight (DW). Log moisture content was recorded as percent water, calculated as (1−(DW/WW)) × 100. We assessed correlations among these three variables using pairwise comparisons. All Pearson's *r* values were ≤ 0.15, indicating no evidence of multicollinearity.

### 2.4 Seasonal Analyses

We first focused on meteorological seasons as the basis of assessing relationships between thermal buffering and rotting log size, decay class, and moisture content. Three summary statistics were used to describe thermal buffering for each focal log. In spring (March 1–May 31) and fall (September 1–November 30), we calculated the average reduction in the daily temperature range recorded by internal iButtons compared to external iButtons, as a measure of the reduction of temperature variability inside logs. In winter (December 1–February 29) when temperatures were lowest, we calculated the average difference between daily low temperatures recorded by internal versus external iButtons as a measure of log buffering of ambient cold temperatures. In summer (June 1–August 31) when temperatures were highest, we calculated the average difference between the daily high temperatures recorded by internal versus external iButtons as a measure of log buffering of ambient warm temperatures. Because our objective was to quantify short‐term thermal buffering dynamics (i.e., reduction of variability, and of extremes) rather than cumulative thermal exposure, we did not use degree‐day metrics, which integrate temperature over time.

Using the aforementioned summary statistics as response variables, we built four linear mixed‐effects models—one for each meteorological season—with log diameter, decay stage, and moisture content, and each of the two and three‐way interactions as fixed effects, and the specific local site and broader forest region in which logs occurred as nested random effects. These analyses were performed using the lme4 package (Bates et al. 2015) in R (R Core Team 2024). We performed model selection using an information‐theoretic approach with the MuMIn package in R (Bartoń 2025). Starting from a global linear mixed‐effects model that included all candidate predictors, we used the dredge() function to generate all possible subsets of fixed effects. Each candidate model was ranked by Akaike Information Criterion (AIC), and Akaike weights were calculated for each model. For each predictor, we computed the sum of Akaike weights across all models in which the predictor appeared using the sw() function. Predictors with a cumulative weight greater than 0.5 were considered influential and retained for the final mixed‐effects model. We then refitted this final model using the lmer() function from the lme4 package. Two logs were much larger than all other logs (i.e., diameters > 50 cm) and preliminary analyses indicated that they were highly influential outliers in linear mixed‐effects models. Accordingly, we discarded these two logs prior to running these analyses.

### 2.5 Analyses of Extreme Temperatures

Thermal tolerance thresholds have a strong impact on where a species can survive and reproduce, and consideration of climatic extremes often improves prediction of species range boundaries (e.g., Zimmermann et al. 2009; Chen and Lewis 2024). Indeed, species distribution models for several eastern United States saproxylic invertebrate taxa have repeatedly identified maximum temperature of the warmest month (BioClim 5) or minimum temperature of the coldest month (BioClim 6) as informative variables (Hyseni and Garrick 2019a, 2019b; Ulyshen et al. 2017; Garrick et al. 2021). Accordingly, here we explicitly analyzed extreme temperatures. To objectively identify extremes, we first obtained publicly available daily temperature data from weather stations that were spatially proximate to local sites within the three forest regions over the 10‐year period preceding the end of our sampling period. For the assessment of extreme highs, we selected temperature datapoints from external iButtons that were above one standard deviation from the mean daily maximum temperature recorded at the nearest weather station. Next, we used the difference between the daily maximum temperatures of paired external and internal iButtons as a measure of rotting log buffering of extreme highs. We then fit a global linear mixed‐effects model predicting buffering of extreme highs as a function of log diameter, decay stage, and moisture content, including all two‐ and three‐way interactions as fixed effects, with weather station from which historical data were retrieved and individual log identify as nested random effects. Model selection followed the same approach as described above. The same approach was used to assess extreme lows. Briefly, temperature datapoints from external iButtons below one standard deviation from the mean daily minimum temperature recorded over the preceding 10 years at the nearest weather station were selected, and rotting log buffering of extreme lows was measured via the difference between the daily minimum temperatures of paired external and internal iButtons. For the model of extreme high temperatures, because no predictor exceeded the cumulative Akaike weight threshold (see Results), we additionally evaluated a simplified model including log diameter as the sole fixed effect, given its a priori ecological relevance and consistent importance across other buffering metrics, and weather station as a random effect.

## 3 Results

### 3.1 Overview of the Empirical Data

Over the duration of this study, there was high attrition of internal and/or external iButton temperature loggers, largely owing to tampering by wildlife (i.e., black bears and racoons, as indicated by bite marks on plastic covers). Ultimately, of the 51 rotting logs in which iButtons were deployed, 31 logs (61%) yielded complete (or nearly complete) size, decay stage, moisture content, and temperature data. Spatially, the 31 focal logs were distributed as follows: 10 across three local sites at Bankhead National Forest, 11 across five sites at Great Smoky Mountains National Park, and 10 across four sites at Shenandoah National Park (Table 1). All local sites were separated by at least 3.8 km (mean among‐site distances: Bankhead National Forest = 16.1 km, Great Smoky Mountains National Park = 27.6 km, and Shenandoah National Park = 45.4 km). Excluding the two largest logs that were considered outliers (see *Seasonal Analyses* in Methods), diameters ranged from 15.0–46.6 cm (mean: 26.63 cm, standard deviation [SD]: 7.39 cm). Decay classes 2, 3 and 4 were well represented, with each of these classes accounting for 25% to 32% of all focal logs. Class 1 was less well represented (14%), and no class 5 logs were included (Appendix 1). Rotting log moisture content was quite high, and varied little (mean: 67.09%, SD: 7.36%). Notably, temperature data were obtained for a relatively short period in summer (mean: 42 days per log, range: 20–62 days), whereas the other three seasons had complete temporal coverage (i.e., 90–92 days per log, depending on the season and year; Appendix 2).

**TABLE 1:** Summary of the geographic locations from which temperature data were collected inside and outside of rotting logs across the Southern Appalachian Mountains.

Forest region	Local site name	No. of logs at local site	Latitude	Longitude	Elevation (m)
BNF	Houston Rec. Area	3	34.12163	−87.29019	203
BNF	Corinth rec. Area	3	34.10303	−87.32437	186
BNF	Cranal Road	4	34.28417	−87.42841	275
GSMNP	Big Witch Overlook	3	35.52537	−83.22394	1281
GSMNP	Cove Creek	2	35.64000	−83.05649	1109
GSMNP	Gunter Cemetery	2	35.77133	−83.21353	587
GSMNP	Greenbrier Road	2	35.73248	−83.41105	454
GSMNP	Little River Gorge Road	2	35.67937	−83.55926	507
SHEN	Jeremy's Run Overlook	3	38.71189	−78.32933	734
SHEN	Franklin Cliffs Overlook	3	38.53670	−78.41842	961
SHEN	Doyles River Overlook	2	38.24756	−78.69287	892
SHEN	Sawmill Run Overlook	2	38.11461	−78.78404	669

*Note:* Given multiple logs per local site, coordinates and elevation are reported here as an average of all logs at a given site (see Appendix 1 for per‐log information).

Abbreviations: BNF, Bankhead National Forest, Alabama; GSMNP, Great Smoky Mountains National Park, Tennessee; SHEN, Shenandoah National Park, Virginia.

**FIGURE 3:**
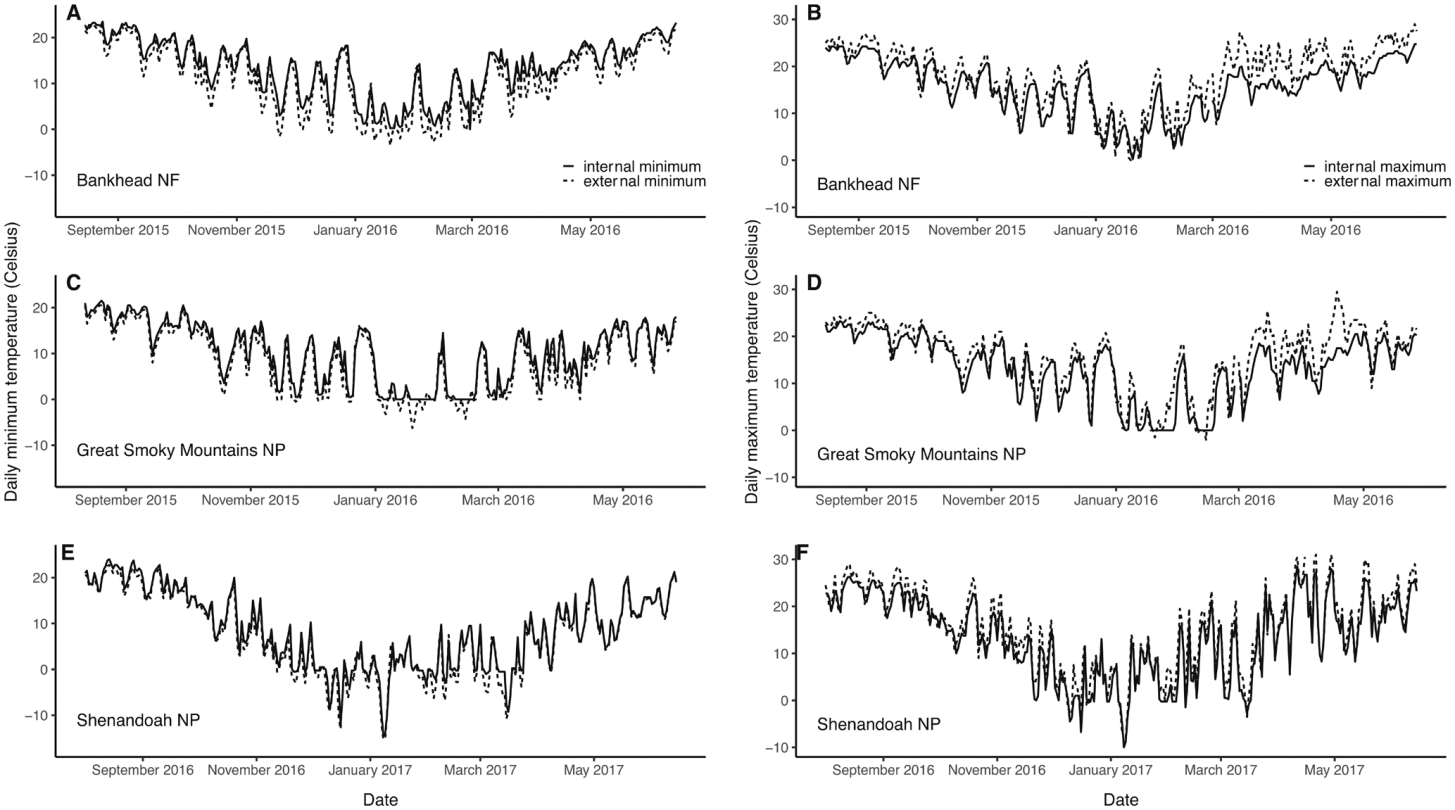
Daily minimum (left) and daily maximum (right) temperatures based on internal (solid lines) and external (broken lines) iButtons over the duration of the study, averaged across 10–11 focal rotting logs within each of three forest regions, as follows: Bankhead National Forest (A, B); Great Smoky Mountains National Park (C, D); and Shenandoah National Park (E, F).

During the ~12‐month temporal sampling period in each of the three forest regions, external iButton temperatures ranged from −16.50°C to 38.75°C (see Appendix 3 for mean ± standard error [SE] and range for internal and external temperatures by forest region and season). Temperatures were typically hottest during the summer, coolest during the winter, and fluctuated greatest during spring. Logs of all diameters and decay classes were generally buffered to some extent against ambient temperatures, with log interiors warmer than ambient temperatures at night, and cooler during the day (Figure 3).

### 3.2 Seasonal Analyses

**FIGURE 4:**
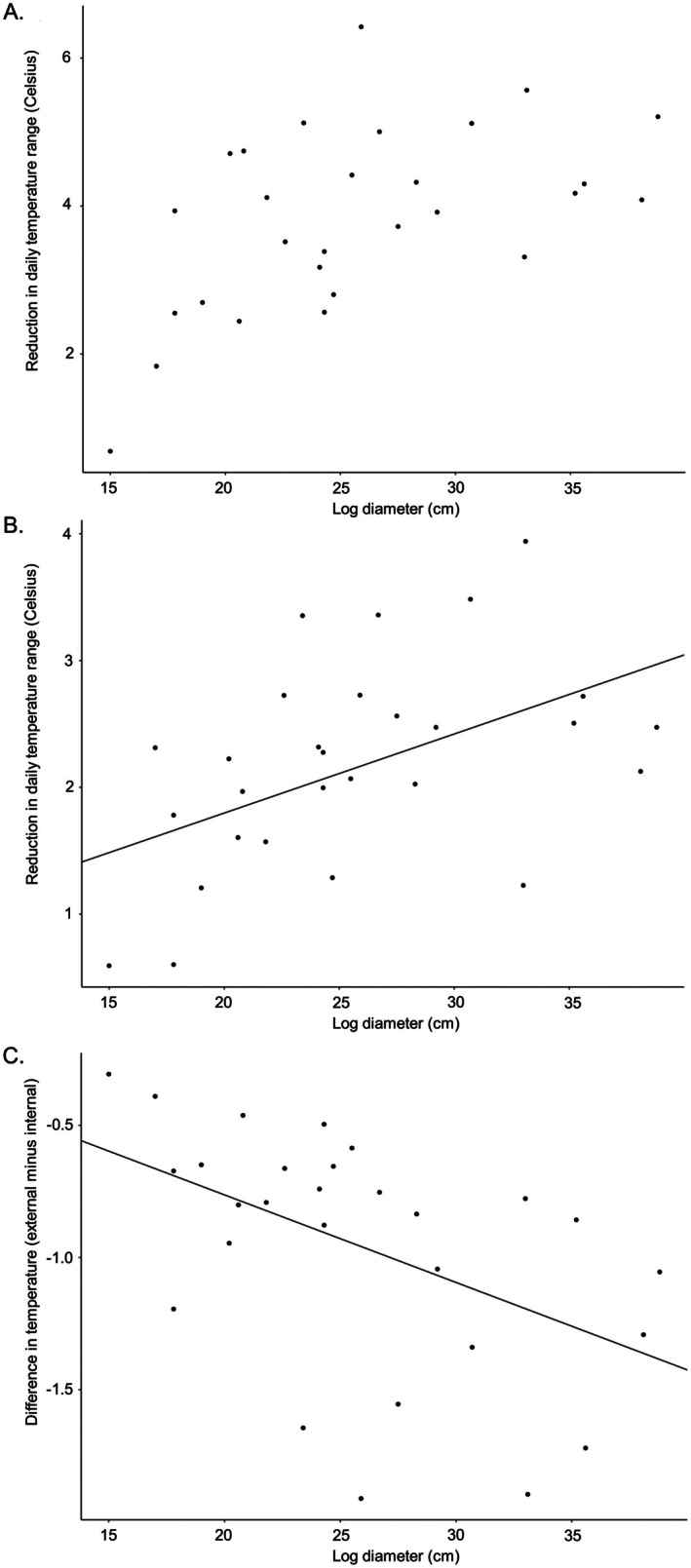
Plots showing the relationship between rotting log diameter and metrics of thermal buffering, for each of the three seasons in which there was significant association between predictor and response variable, as identified in linear mixed effect models. (A) spring model and (B) fall model, both showing that larger logs experience a reduced average daily temperature range; (C) winter model, showing that larger logs experience warmer average daily internal minimum temperatures (log‐transformed to satisfy the assumption of normally distributed residuals when running the linear mixed‐effect model).

Based on AIC model selection, log diameter was chosen as the sole predictor in the final model for three of the four seasons (Table 2). Diameter was positively correlated with the magnitude of reduction in temperature variability in both spring (ß = 0.103, *t* = 3.617, *p* = 0.002; Figure 4a) and fall (*ß* = 0.062, *t* = 4.235, *p* < 0.001; Figure 4b). In winter, log diameter was positively correlated with increased average minimum log interior temperature (*ß* = 0.039, *t* = 3.530, *p* = 0.002; Figure 4c). However, in summer neither log diameter nor any of the other variables considered were significant predictors of thermal buffering of warm temperatures within log interiors. The lack of a significant effect in summer should be interpreted with caution, as this season was represented by a shorter sampling period (~2 months vs. ~3 months for other seasons).

**TABLE 2:** Sum of Akaike Weights for each predictor in all four of the seasonal linear mixed‐effect models.

Model predictor(s)	Summer	Fall	Winter	Spring
Diameter	0.13	**0.89**	**0.55**	**0.77**
Percent water	0.05	< 0.01	< 0.01	0.02
Decay class	< 0.01	< 0.01	0.02	0.29
Diameter: percent water	< 0.01	< 0.01	< 0.01	< 0.01
Diameter: decay class	< 0.01	< 0.01	< 0.01	< 0.01
Decay class: percent water	< 0.01	< 0.01	< 0.01	< 0.01
Diameter: decay class: percent water	< 0.01	< 0.01	< 0.01	< 0.01

*Note:* Response variables were: Reduction of average daily maximum temperatures (summer), increase of average daily minimum temperatures (winter), or decrease in the range of temperatures experienced in (spring and fall). Only the predictor(s) with a sum of Akaike Weights > 0.5 (shown in bold) were included in our final models.

### 3.3 Analyses of Extreme Temperatures

**FIGURE 5:**
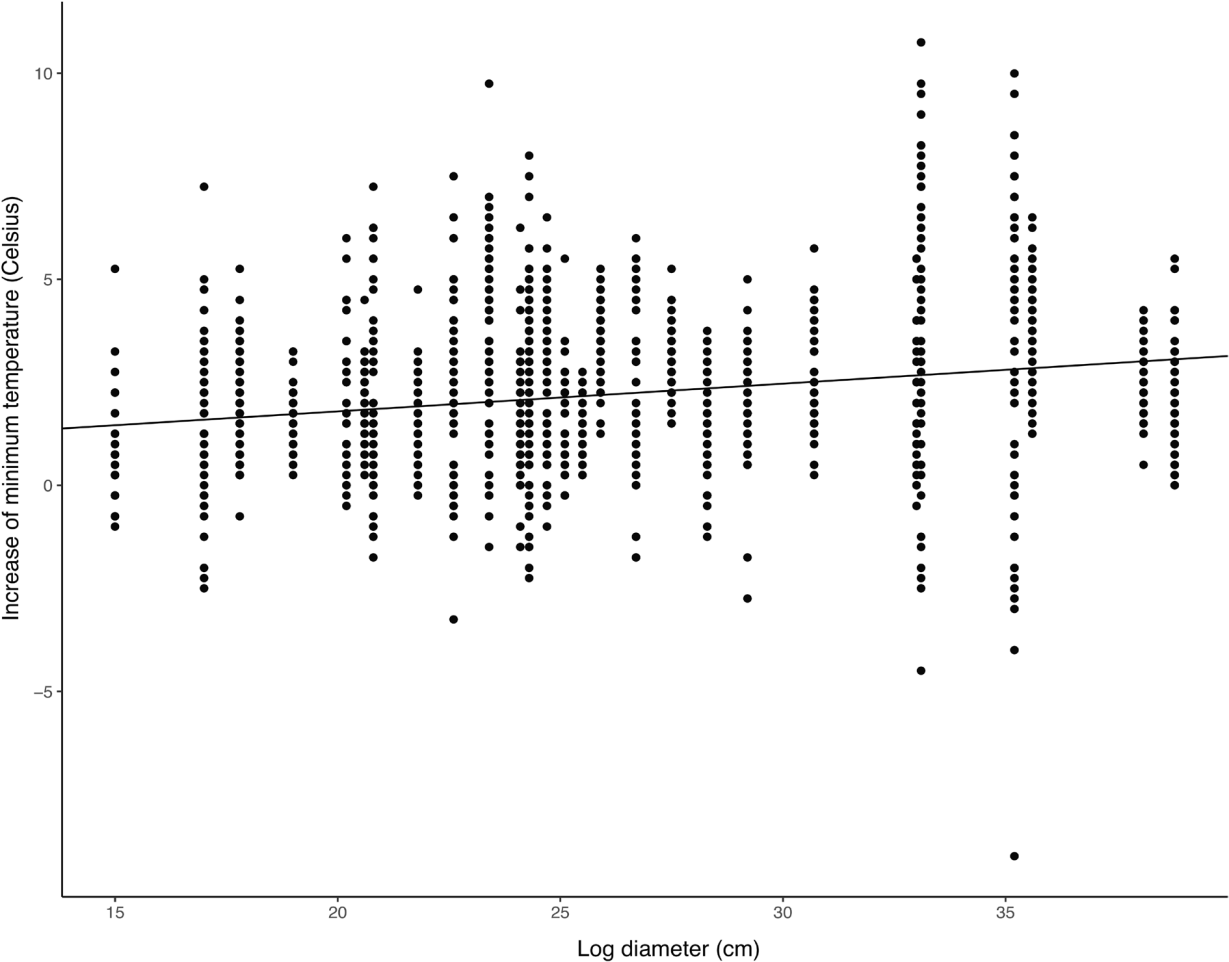
Relationship between rotting log diameter (predictor) and magnitude of increase in internal temperature on extreme cold days (response), showing that larger logs experienced warmer internal minimum temperatures.

Based on AIC model selection, log diameter was retained as the sole fixed effect in the final model for buffering of extremely low temperatures. In contrast, none of the predictors in the model for extremely high temperatures exceeded a cumulative Akaike weight of 0.5 (Table 3), indicating limited support for any single predictor. We therefore present results from the simplified model including diameter as the sole fixed effect for extreme high temperatures. We found that diameter was a significant predictor of higher internal minimum temperatures of rotting logs on extreme cold days, with larger logs providing more buffering from extreme lows (*ß* = 0.033, *t* = 7.089, *p* < 0.001; Figure 5). In contrast, log diameter was not correlated with reduced internal maximum temperatures on extreme hot days (*t* = 0.910, *p* = 0.364).

**TABLE 3:** Sum of Akaike Weights for each predictor in the two linear mixed‐effects models of thermal buffering in rotting logs under extreme temperatures.

Model predictor(s)	Maximum temperatures	Minimum temperatures
Diameter	0.04	**0.80**
Percent water	0.02	0.02
Decay class	0.08	0.33
Diameter: percent water	< 0.01	< 0.01
Diameter: decay class	< 0.01	< 0.01
Decay class: percent water	< 0.01	< 0.01
Diameter: decay class: percent water	< 0.01	< 0.01

*Note:* Response variables were: The difference between internal and external maximum temperatures on extremely hot days, and the difference between internal and external minimum temperatures on extremely cold days. Only the predictor(s) with a sum of Akaike Weights > 0.5 (shown in bold) were included in our final models.

## 4 Discussion

This study represents one of just a handful of investigations that have assessed thermal buffering of rotting logs in temperate forests, and of these, it is the only one in which log diameter was measured as a continuously varying characteristic (cf. treated as a large vs. small categorical variable defined a priori, or was uniform or unquantified; see Kluber et al. 2009; Romo et al. 2019; Barnes et al. 2022; Lawhorn and Yanoviak 2022). In boreal forests where buffering of cold stress during winter may be particularly important, log diameter has more commonly been explicitly considered as a continuous variable (e.g., Pouska et al. 2016, 2017; Lindman et al. 2022; Schreiber et al. 2025). That said, it is rare for studies to combine broad geographic sampling with collection of temperature data measured both inside and outside of logs, such that buffering metrics can be calculated. Even fewer span all four seasons, and incorporate information on log decay class and moisture content. Below, we draw findings from our observational case study to address the primary questions of how large, decayed, and moist must rotting logs be to act as thermally buffered microhabitats in natural temperate mixed pine‐oak forests in the eastern United States. Given the limited availability of thermal tolerance data for saproxylic invertebrates (but see Cox et al. 2020; Barnes et al. 2022; Lawhorn and Yanoviak 2022), the detailed characterization of log microclimates presented here—including temperature extremes, variability, and short‐term ramp rates—provides a critical foundation for future functional ecology studies. In particular, these data can inform experimental design by defining ecologically realistic thermal regimes, which are essential for accurately assessing organismal responses to temperature in wood‐associated habitats.

### 4.1 How Large Do Rotting Logs Need to Be to Provide Thermal Buffering?

We found that larger diameter logs generally have greater thermal insulating capacity than smaller logs (Table 2). This was evident in three of four seasonal analyses, supported either by a decrease in the range of temperatures experienced in spring and fall (Figure 4a,b), or an increase in average daily minimum temperatures in winter (Figure 4c). It was also evident in our analysis of extreme temperatures, where larger rotting logs had higher internal minimum temperatures on very cold days (Figure 5). Other studies have also reported more pronounced reductions in daily temperature fluctuations inside larger logs compared to smaller logs (e.g., Pouska et al. 2016; Lindman et al. 2022). Qualitatively, our data indicate that rotting logs of at least 25–30 cm in diameter can be expected to provide a considerable amount (e.g., 4°C) of thermal buffering of diurnal temperature range during spring months when temperature was most variable (Figure 4a). Logs of this size are also strong candidates for providing at least a modest amount (e.g., 1°C) of insulation against cold in winter (Figure 4c). Indeed, there may not be considerable added benefit of logs that are much larger than this. In a study by Schreiber et al. (2025) in which most logs (> 65%) were quite large (> 30 cm), no major impact of log diameter on thermal buffering was detected, potentially indicating a plateau of thermal buffering. Several studies focused on saproxylic invertebrate biodiversity have classified “large logs” using a threshold that closely matches the aforementioned size required for considerable buffering of temperature range and modest buffering of minimum temperatures (e.g., Brin et al. 2011; > 20 cm for pine, and > 30 cm for oak). However, other conservation‐focused studies have used higher thresholds (e.g., Grove 2002b; > 40 cm for tropical rainforest logs; Yee et al. 2006: > 100 cm for cool temperate wet sclerophyll *Eucalyptus* logs). To determine if there really are diminishing returns on thermal buffering capacity of logs with diameters > 25–30 cm, additional research is needed.

Somewhat surprisingly for temperate forests, our data indicated that large diameter logs showed no significant thermal insulation from summer heat. This finding may reflect type II error in our summer seasonal analysis and the analysis of extreme hot days, owing to the small number—and potentially unrepresentative nature—of datapoints collected over summer. That said, in a temperate coniferous forest in the Pacific Northwest of the United States, Kluber et al. (2009) compared internal and external temperatures in small and large logs (classified a priori) during the warmest month of summer and found no significant differences. Likewise, notwithstanding the considerable differences in landscape setting of a native pine forest in Sweden, Lindman et al. (2022) reported that in comparison to external temperatures, internal log microclimate was slightly warmer in summer. Thus, it is worth considering the broader implications of the aforementioned result, as it may reflect a real biological pattern. Rotting logs serve as important refugia from heat for saproxylic invertebrates (Schmuki et al. 2007; Woodman et al. 2007; Cox et al. 2020; Barnes et al. 2022; Lawhorn and Yanoviak 2022). Eggs and juvenile life stages are at greatest risk of lethal heat exposure (e.g., larval holometabolous insects are soft‐bodied and most dispersal‐limited), and for the most common North American saproxylic beetles, life cycles often take > 1 year to complete (e.g., *Odontotaenius disjunctus* , Bibbs et al. 2010; *Lucanus elaphus* , Ulyshen et al. 2017). Thus, phenological escape from summer heat is unlikely. Accordingly, behavioral responses such as moisture‐seeking (e.g., tunneling deeper within a log, or closer to the soil‐log interface), or acclimation (perhaps facilitated by slower temperature ramp rates within logs) may be particularly important. Also, of the few studies that report CTmax of saproxylic invertebrates, values are generally > 40°C (Barnes et al. 2022; Lawhorn and Yanoviak 2022) which were rarely approached in our study, whereas lower temperature thresholds (e.g., CTmin ~4°C in at least one species; Cox et al. 2020) fall within the range of ambient conditions that we observed. As noted above, this suggests that buffering of cold extremes may be more biologically relevant than buffering of high temperatures, consistent with our results. More generally, thermal gradients within logs—quantified usingdata loggers placed at different depths (e.g., Romo et al. 2019; Lawhorn and Yanoviak 2022)—warrant further investigation. Such work would improve understanding of within‐log heterogeneity, including localized zones of enhanced buffering that log‐restricted ectotherms could exploit to optimize conditions through very short‐distance movements, perhaps on the order of a few centimeters.

### 4.2 How Decayed Do Rotting Logs Need to Be?

Log decay stage was not identified as a potential predictor of thermal buffering in our study. Given that only four classes within the five‐class scale were represented, it is possible that the dataset had low statistical power. A broader limitation of this and other studies is that the classification process itself is subjective. We adopted Barclay et al.'s (2000) scheme owing its focus on cross‐section profiles, as this could be readily applied to the experimentally removed segment of each log containing the internal and external iButtons. Alternative approaches exist (e.g., written descriptions of five‐class scales by Cline et al. 1980 and Sollins 1982), but they are applied to the whole log, which can be challenging given that these units often have characteristics of multiple decay classes (Pyle and Brown 1999). More objective classifications could be facilitated by quantifying wood density or C:N ratio, as both are negatively correlated with log decay class (Hyde et al. 2011; Maser et al. 1988).

Regarding the potential relationship between thermal buffering and decay stage, Pouska et al. (2016) considered a similar range of log classes (i.e., stages 2–5, cf. stages 1–4 here) and found that temperature stability increased with the progression of wood decay. Other studies have qualitatively compared a class 1 versus class 3 log (*n* = 1 each, over a single day in summer; Barnes et al. 2022; Lawhorn and Yanoviak 2022). Notwithstanding the limited sample size and temporal coverage, those authors reported that the average temperature inside the class 1 logs were higher (up to ~10°C) than class 3 logs, and exceeded that of ambient air (class 3 log average internal temperatures were below ambient). This suggests an initial barrier to colonization early in the decomposition process that puts a premium on CTmax of pioneer taxa (i.e., free‐living wood‐digesting fungi and other microbes, termites, woodboring beetles; Ulyshen 2016). Indeed, termites are quite resilient to dry conditions, as they can persist in subterranean nests (Ulyshen 2014). Notably, the soil subsurface becomes thermally buffered from extreme temperatures when CWD is in close proximity, even if the CWD is a class 1 log (Goldin and Hutchinson 2015). That said, our understanding of thermal properties of stage 5 logs remains limited, and studying advanced stages of wood decomposition has been identified as one of the most pressing needs in deadwood ecology (Seibold et al. 2015).

### 4.3 How Moist Do Rotting Logs Need to Be?

We considered rotting log moisture content as a potential predictor in our models, but it was not identified as having a significant effect on thermal buffering. Pouska et al. (2016) reported that as rotting log water content increases, the interior microclimate experiences warmer minimum temperatures, cooler maximums, and reduced fluctuations. Likewise, water content typically increases with log diameter (Lindman et al. 2022), making it somewhat surprising that our observed significant effects of diameter on temperature were not also reflected in the moisture content data. That said, moisture content was consistently high, and varied little (mean: 67.09%, SD: 7.36%), making any effects difficult to detect in the absence of larger sample sizes and/or greater measurement precision. Nonetheless, our findings underscore the potentially important role that CWD plays as a water reservoir for biota that obligately or facultatively use deadwood microhabitats, particularly during drought.

Generally, water content in logs increases until it reaches decay class 4, and thereafter it either stabilizes or declines (Maser et al. 1988). On a finer temporal scale, however, moisture content within a rotting log can be quite dynamic, such that a single point estimate may be strongly influenced by the most recent rainfall. Green et al. (2022) measured volumetric water content within a decay class 3 log at 15‐min intervals over a 4‐month period spanning summer and fall. The authors found that rainfall events were indeed the primary driver, and that rotting log moisture content covaried with that of the soil surface. Accordingly, our single timepoint measure of gravimetric moisture content likely provides only a coarse snapshot of conditions and may not reflect moisture regimes over the full study period. This limitation may explain why moisture content was not a significant predictor of thermal buffering in our analyses, and thus our results should be interpreted in this context. Use of sensors capable of continuously measuring volumetric water content over time (as in Pouska et al. 2016, and Green et al. 2022) would provide a more appropriate basis for evaluating the role of moisture in shaping thermal dynamics within rotting logs.

### 4.4 Practical Management Recommendations

Ongoing forest fragmentation and reductions in the spatial and temporal continuity of coarse woody debris (CWD) represent major threats to saproxylic arthropods (Schiegg 2000; Seibold et al. 2015), driven by activities such as land clearing, clear‐cutting, and firewood collection. Interventions that cater to the needs of saproxylic organisms are well‐established in Europe. In addition to silvicultural practices on private and public land such as selective logging to create uneven‐aged stands, at a local scale, at least five management interventions could be considered for enhancing the quality, quantity and connectivity of deadwood. These include: (1) increasing contact between new CWD and the forest floor to facilitate fungal colonization from soil (Garrett et al. 2010); (2) disrupting the structural integrity of new CWD to facilitate fungal colonization via airborne spores (Berglund et al. 2011); (3) felling selected large‐diameter snags to increase the quantity of logs with high moisture and thermal buffering capacity (Michaels and Bornemissza 1999); (4) killing selected trees, either immediately by girdling (i.e., a deep cut around the trunk that removes xylem, preventing transpiration), or slowly by ring‐barking (i.e., a shallow cut that removes bark and cambium, causing starvation over months/years; Moore 2013); and (5) “veteranizing” selected living trees (i.e., unconventional arboriculture techniques that generate markedly ‘ancient’ characteristics, irrespective of chronological age) to enhance future CWD inputs with a long lag‐time (Lonsdale 2013). With respect to thermal buffering, our data suggest that focusing on logs, snags and/or living trees with a diameter of at least 25–30 cm could be particularly valuable deadwood microhabitats.

## Author Contributions

**Ryan T. Phillips:** conceptualization (supporting), data curation (lead), formal analysis (lead), writing – original draft (supporting), writing – review and editing (supporting). **Ryan C. Garrick:** conceptualization (lead), data curation (supporting), formal analysis (supporting), funding acquisition (lead), writing – original draft (lead), writing – review and editing (lead).

## Funding

This work was partially funded by grants from the Bay and Paul Foundations, Conservation and Research Foundation, Eppley Foundation for Research, National Geographic Society, and the Washington Biologists’ Field Club.

## Conflicts of Interest

The authors declare no conflicts of interest.

## Data Availability

Raw temperature data from iButtons, and associated metrics of thermal buffering, are available via the following Dryad Repository entry: https://doi.org/10.5061/dryad.02v6wwqhn.

## Appendix 1 Log‐Specific Information for the 31 Rotting Logs Used in Our Analyses. Log IDs Correspond With Raw Temperature Data and Thermal Buffering Metrics Available in DRYAD. Forest Region Abbreviations Follow Those Used in Table 1. Gray/White Shading Groups Logs That Occur at the Same Local Site. Temporal Coverage of iButton Data From Each Log Is Shown in Appendix 2

**Table:**

Log ID	Forest region	Local site name	Latitude	Longitude	Elevation (m)	Diameter (cm)	Water (%)	Decay class
C01	BNF	Houston Rec. Area	34.12192	−87.29025	198	19.0	75.52	4
C02	BNF	Houston Rec. Area	34.12176	−87.29032	202	28.3	73.47	4
C12	BNF	Houston Rec. Area	34.12120	−87.29002	208	25.9	61.67	3
C13	BNF	Corinth Rec. Area	34.10336	−87.32465	190	30.7	68.83	3
C14	BNF	Corinth Rec. Area	34.10343	−87.32438	186	25.5	68.76	3
C15	BNF	Corinth Rec. Area	34.10230	−87.32407	181	17.7	61.69	1
C57	BNF	Cranal Road	34.28418	−87.42854	275	27.5	78.27	1
C58	BNF	Cranal Road	34.28415	−87.42840	269	38.1	64.34	4
C59	BNF	Cranal Road	34.28410	−87.42831	279	20.6	66.96	1
C60	BNF	Cranal Road	34.28423	−87.42839	276	35.6	63.37	1
C25	GSMNP	Big Witch Overlook	35.52532	−83.22337	1272	24.7	63.64	2
C26	GSMNP	Big Witch Overlook	35.52518	−83.22374	1285	61.0	70.67	2
C28	GSMNP	Big Witch Overlook	35.52561	−83.22472	1286	33.0	66.41	4
C29	GSMNP	Cove Creek	35.64004	−83.05638	1109	15.0	68.89	2
C31	GSMNP	Cove Creek	35.63996	−83.05661	1109	50.8	69.19	2
C39	GSMNP	Gunter Cemetery	35.77123	−83.21352	592	17.8	37.60	3
C40	GSMNP	Gunter Cemetery	35.77142	−83.21355	582	25.1	69.50	NA
C41	GSMNP	Greenbrier Road	35.73247	−83.41083	441	38.8	67.71	2
C44	GSMNP	Greenbrier Road	35.73249	−83.41127	466	21.8	72.67	4
C45	GSMNP	Little River Gorge Road	35.67947	−83.55922	504	29.2	78.76	2
C47	GSMNP	Little River Gorge Road	35.67926	−83.55930	510	24.1	71.57	4
C65	SHEN	Jeremy's Run Overlook	38.71128	−78.32976	735	22.6	72.06	2
C67	SHEN	Jeremy's Run Overlook	38.71269	−78.32880	735	46.6	65.16	2
C68	SHEN	Jeremy's Run Overlook	38.71169	−78.32944	733	23.4	62.72	3
C69	SHEN	Franklin Cliffs Overlook	38.53701	−78.41846	963	20.8	64.06	3
C70	SHEN	Franklin Cliffs Overlook	38.53679	−78.41842	963	24.3	70.37	4
C72	SHEN	Franklin Cliffs Overlook	38.53630	−78.41837	956	35.2	71.32	3
C73	SHEN	Doyles River Overlook	38.24725	−78.69313	893	33.1	64.67	4
C74	SHEN	Doyles River Overlook	38.24787	−78.69261	890	26.7	62.26	2
C81	SHEN	Sawmill Run Overlook	38.11437	−78.78395	667	24.3	65.89	2
C85	SHEN	Sawmill Run Overlook	38.11485	−78.78414	671	17.0	67.46	2

## Appendix 3 Summary Statistics (Mean ± SE and Range) of Temperatures (°C) Recorded by Internal and External iButtons, Stratified by Forest Region and Season. Forest Region Abbreviations Follow Those Used in Table 1

**Table:**

	BNF		GSMNP		SHEN	
	Internal	External	Internal	External	Internal	External
Spring						
Range	2.25 to 26.75	0.75 to 38.50	−0.50 to 26.25	−3.00 to 38.50	−9.25 to 28.50	−10.75 to 38.75
Mean (SE)	16.00 (0.06)	16.74 (0.09)	11.21 (0.07)	11.83 (0.08)	10.37 (0.09)	10.73 (0.11)
Summer						
Range	15.75 to 30.00	15.00 to 38.25	11.50 to 24.50	11.25 to 25.00	10.50 to 28.00	8.75 to 31.75
Mean (SE)	23.92 (0.04)	24.22 (0.05)	19.25 (0.06)	18.90 (0.07)	20.16 (0.05)	20.28 (0.06)
Fall						
Range	0.25 to 27.75	−2.00 to 33.50	−3.00 to 26.25	−6.25 to 25.00	−0.75 to 26.00	−5.25 to 29.50
Mean (SE)	16.82 (0.07)	16.87 (0.08)	12.94 (0.07)	12.70 (0.07)	12.39 (0.08)	12.47 (0.09)
Winter						
Range	−1.25 to 21.25	−4.50 to 30.00	−16.00 to 19.75	−16.50 to 20.75	−15.00 to 21.25	−16.00 to 29.25
Mean (SE)	8.34 (0.07)	8.37 (0.09)	3.69 (0.07)	3.66 (0.08)	1.96 (0.06)	2.21 (0.08)
